# Strategic Variation in Male Courtship Persistence in the Butterfly *Eurema hecabe*

**DOI:** 10.1002/ece3.72521

**Published:** 2025-12-11

**Authors:** Sanni A. Silvasti, Darrell J. Kemp

**Affiliations:** ^1^ School of Natural Sciences Macquarie University Sydney Australia

**Keywords:** courtship, mate search tactics, teneral mating, time investments, visual search

## Abstract

The males of many insect species have experienced intense sexual selection for the ability to swiftly locate sexually receptive females. This is thought to have engendered the evolution of highly efficient mate location strategies, and to favour flexibility in male behaviour as commensurate with the potential net payoff in terms of securing a mate. In the common grass yellow butterfly (*Eurema hecabe*), males visually search for receptive females by patrolling in and around patches of the larval host plant. Females of this weakly polyandrous species are sexually receptive immediately upon eclosion, and then once or possibly twice during adulthood. For the males, the likelihood of achieving copulation is very high when they encounter a newly eclosed, immobile female, but low when they approach older, pre‐mated females, who are generally seeking to oviposit. Here we show that *E. hecabe* males optimise their chances of copulation by allocating their mate search efforts according to the likely availability of receptive females. Males engage in a dedicated search for freshly emerged (teneral) females around the peak time of pupal eclosion (early morning) then shift to lengthier courtships of older, flying females (late morning). The males appear to continue the search for teneral matings alongside the courting of mature females in the late mornings, so the primary behavioural shift concerns the length of time males are willing to invest in courting flying females. These findings support the general expectation for flexibility in male search tactics as a function of female receptivity.

## Graphical abstract

Male Eurema hecabe butterflies adjust their mate‐searching strategy to match female receptivity. Early in the morning, when most females eclose, males focus on locating freshly emerged, immobile females; later, they invest more time courting older, flying females while still searching for tenerals. These findings support the general expectation for flexibility in male search tactics as a function of female receptivity.

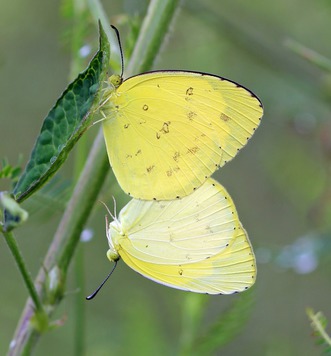

## 1 Introduction

The males of many insect species are subject to strong sexual selection to maximise their success in fertilising female gametes (Thornhill and Alcock 1983). Although the specifics vary across groups, a widespread target of sexual selection is the ability for males to be the first to locate receptive females. The problem faced in doing so is non‐trivial because the potential search environment typically far exceeds the limit of any individual male's sensory capacity. For this reason, male mate location in butterflies is typified by strategies based upon different searching tactics (Rutowski 1991). In some species, males establish and aggressively defend territories in sites that are favourable for encountering females, such as prominent flight paths or patches of scarcely distributed larval hostplant (D. Kemp 2018; Kemp and Wiklund 2001). Such males perch on vegetation or on the ground and take flight to inspect moving objects that could be bypassing females or intruding males. However, in most butterflies, mate search involves a more mobile patrolling tactic (Rutowski 1991). In patrolling behaviour, rivalry for mates plays out as scramble competition (*sensu* Andersson 1994), where success is defined by the ability to most effectively locate receptive females. Patrolling males invest a significant proportion of their time in a visual search for potential mates and, at a fast pace, inspect any object fitting the scope in terms of colour and size.

Patrolling has particular utility in species where females are sexually receptive immediately upon eclosion. The males of such species search larval host plant sites for pre‐emerging pupae and/or recently emerged, immobile females, which are mated during or immediately following emergence (Estrada and Gilbert 2010; Kato and Nakane 1989; Silberglied and Taylor 1978; Thurman et al. 2018). In cases where mating occurs during eclosion (or in some cases immediately prior to eclosion), the phenomenon is referred to as pupal mating (Deinert et al. 1994; Gilbert 1975, 1976). This is common in *Heliconius* butterflies (Beltran et al. 2007; Deinert et al. 1994). If mating occurs immediately after eclosion, whilst the female's wings are yet to flatten or are still soft, the mating is referred to as ‘teneral mating’ (see Thurman et al. 2018). In both cases, females are not thought to possess the ability to reject a courting male (Silberglied and Taylor 1978; Thurman et al. 2018). Such females offer a potentially high fitness payoff for males, as the likelihood of courtships leading to mating among adult butterflies is generally low (Craw 1975; Lundgren and Bergström 1975; Rutowski 1984; Shapiro 1970; Thornhill and Alcock 1983).

In the family Pieridae, the females of some species are polyandrous but also receptive upon pupal emergence (Kato and Nakane 1989; Silberglied and Taylor 1978). This presents the opportunity for males to target two potential sources of receptive mates; namely, immobile teneral females versus free‐flying adult females. The common grass yellow butterfly (*Eurema hecabe*) is a species where both freshly emerged and mature females can be receptive (Hiroki and Obara 1997; Kato 1986; D. J. Kemp 2008). Owing to the features of host plant ecology, both sources of females routinely exist at the same locations at the same time and often reach remarkable population densities (D. J. Kemp 2008; Ramana et al. 2003). Most teneral females are available at the primary eclosion time, which is reported to occur in the morning, whereas adult females tend to be found ovipositing throughout the day, starting from late mornings (Narender 2006). Males of *E. hecabe*, much like other Pierid butterflies (such as *Colias eurytheme* ; Kingsolver 1983), do not exhibit pupal or resource guarding and will instead invest almost all their time patrolling in search of receptive females (Kato 1986; Kato and Nakane 1989).

We hypothesise that male *E. hecabe* should shift between searching for newly emerged females and courting older females in accordance with the likely availability of sexually receptive mates. Specifically, we expect males to participate in scramble competition for teneral matings early in the morning, when adults are eclosing, and shift to searching for and courting older ovipositing females as the morning progresses. Because the time available to search for emerging females is at a premium, we predict that males should perform briefer investigations of potential mates early in the morning, and tend not to engage in protracted courtships (i.e., males should only have brief ‘approach and reject’ interactions with unreceptive females). Later in the morning, males should be more motivated to invest time in harassing rejective females and staying in pursuit of a flying female, given that courtship persistence is associated with mating success (Papke et al. 2007). We therefore predict that in late mornings, male–female interactions should last longer and escalate to aerial pursuit flights, and that the duration of these pursuits should increase across the morning hours.

We aimed, first of all, to quantify the diel timing of pupal emergence in this species (which has been stated to occur during morning hours in Indian *E. hecabe* (Narender 2006) and most likely very early in the morning, as observed in similar relatives such as *C. eurtheme*; (Silberglied and Taylor 1978)), and then to test the predictions as outlined above for male mate search behaviour throughout the morning at a field mate encounter site.

## 2 Methods

### 2.1 Courtship Behaviour of *E. hecabe*

As mentioned above, females of *E. hecabe* are receptive for teneral mating immediately after eclosion, and the majority of them are likely mated before they gain full mobility (Hiroki and Kato 1996; Hiroki and Obara 1997; Kato 1986; Shapiro 1970). Courtship of mobile adult females starts with the male approaching to within close proximity (ca. < 100 mm) of an ovipositing or foraging female. In virtually all cases, the female will then attempt to evade the male, which establishes a pursuit that may escalate into a rapid zig‐zag flight manoeuvre that ascends many metres (Rutowski 1978; Shapiro 1970). These flights may function as an avenue for females to assess male fitness in terms of pursuit performance and durability (*sensu* Edmunds 1969). Pursuit flight length (i.e., courtship persistence) is correlated with mating success (Papke et al. 2007) and can be an indication of the male reproductive potency, as recently mated males have been shown to have reduced courting persistence and lower quality ejaculates (Rutowski 1979). These interactions, however, rarely end in a successful copulation, with most terminating when the male evidently loses sight of the female or gives up the pursuit.

### 2.2 Quantifying the Timing of Pupal Emergence

We observed the timing of pupal eclosion using a high‐density glasshouse‐housed population of *E. hecabe* in November 2024. This population was founded several months earlier by females captured from a North Queensland study site. We started observations at 6 am and checked the glasshouse at least every 30 min. The observation start time was based on the literature (Hannam et al. 2018; Narender 2006; Sencio and Rutowski 2019) on butterfly emergence times, which suggest that eclosion often starts at first light (sunrise occurred at 5.40 am in Sydney at the time of observations). Newly emerged adults take approximately an hour to harden their wings, so we could be confident that early emerging individuals would still be immobile and perched upon or adjacent to their empty pupal case. The counted individuals were removed from the glasshouse to ensure that each was only recorded once.

### 2.3 Behavioural Observations

Behavioural experiments and observations on *E. hecabe* were undertaken in three different field sites within a 5‐km radius from Port Douglas (16.4840° S, 145.4623° E), Northern Queensland on 22 February 2023 and 9–12 March 2023. This timing coincides with the tropical wet season and the peak period of reproductive activity in *E. hecabe*, with population densities at localised patches of host plant often reaching extreme levels (D. J. Kemp 2008). Behaviour was recorded using two GoPro 8 cameras set at 1080p resolution and a 60fps frame rate, placed 6 m apart and aimed at the edge of a large patch of larval hostplant ( *Aeschynomene indica* ). We recorded for a 15‐min period at the beginning of each hour, starting from 7 am until 12 pm. The set of hourly recorded 15‐min video clips from each sampling day was later analysed for behaviour (see below).

Over the four sampling days in March we also visually scanned the field site for male–female interactions and timed their duration (to the nearest 1 s). Interactions were classified as either ‘approach/rejections’ or ‘pursuits’. A typical approach/rejection interaction consisted of a male approaching and inspecting a perching female for a period of time before departing. Females in these interactions frequently discouraged males via rejection behaviours such as wing flicking, rotating their body away from the male or by adopting the classic pierid refusal posture (where a female opens her wings and raises the tip of the abdomen above body axis; (Shapiro 1970; Silberglied and Taylor 1978)). Pursuits, by contrast, consisted of a male approaching a flying female (or harassing a perching female into flight) and then pursuing her in an aerial chase. These pursuits often escalated into ascending rapid zig‐zag flights (Rutowski 1978; Shapiro 1970). We defined the beginning of an interaction as the point where a male approached to within 100 mm of a female, and considered the end of the interaction as when the two butterflies separated and thereafter flew independent trajectories.

### 2.4 Dummy Experiments

**FIGURE A1:**
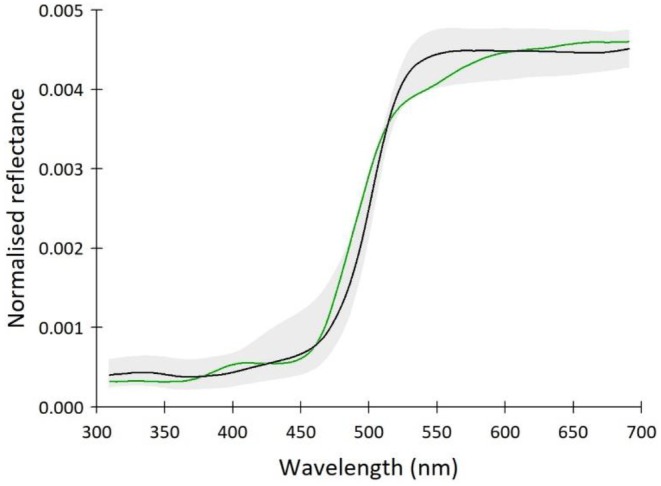
Spectral measurements of the female dummy colour (green line) and the average for the ventral wing of ten *Eurema hecabe* females (solid black trace, range in halftone). Spectra were captured using an Ocean Optics USB‐4000 reflectance spectrometer, with sample illumination provided by a PX‐2 xenon light source (see Kemp and Wedell 2024).

Dummy experiments were performed concurrently with behavioural observations over the four March sampling days (dates as given above). Dummies were designed to resemble perching female *E. hecabe* (Kemp and Macedonia 2007). They consisted of 2.2 × 2.7 cm rounded paper triangles coloured with acrylic paint (Taubmans ‘Lady Lime’, #efd531) that matched the typical female ventral colouration. This match was verified in relation to the ventral colour of 10 undamaged females (captured from the glasshouse) via spectrometry (Figure A1 in Appendix 1).

**FIGURE A2:**
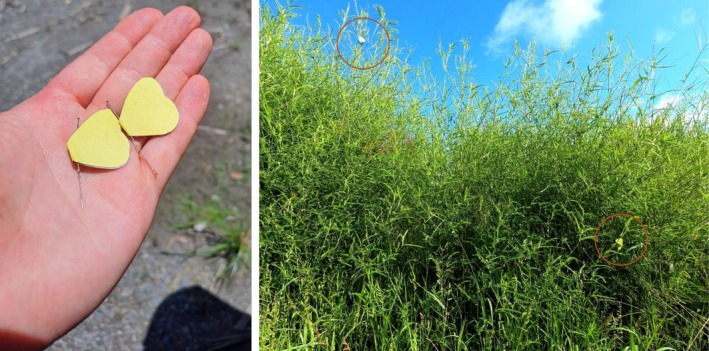
Female dummies used in the experiment. The dummies were pinned on the hostplant vegetation edge, one at the canopy and the other approximately 30 cm above the ground.

At the start of each experimental day we pinned two dummies to the host plant vegetation, one at the top of the vegetation and the other approximately 30 cm above the ground (Figure A2 in Appendix 1). The dummies were filmed using a GoPro 8 camera for 15‐min segments on the hour between 7 am and 12 pm. We subsequently analysed the footage for male approaches to the dummies and timed the durations of each interaction (to the nearest 1 s).

### 2.5 Video Scoring of Butterfly Behaviour

**FIGURE A3:**
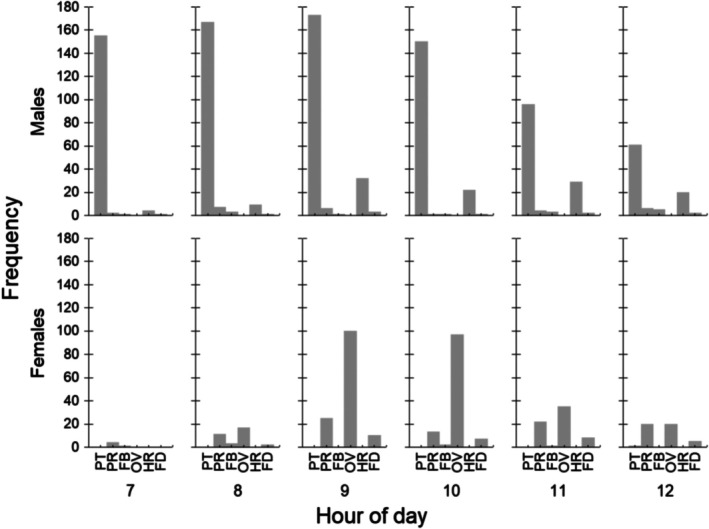
Frequencies of video‐scored behaviours in wild males and females per hour. Behaviour abbreviations are FB, flyby; FD, feeding; HR, harassment; OV, ovipositing; PR, perching and PT, patrolling. Harassment includes both approach/rejection and pursuit flight behaviours.

Butterfly behaviour was transcribed from the video by a single scorer who was not involved in the experimental design or fieldwork, and was naive to the hypothesis being tested. Footage from the two GoPro 8 cameras was used to score butterfly behaviour using an ‘instantaneous scan sampling’ procedure (Bateson and Martin 2021). This involved inspecting the first 10–15 s of every minute of footage and classifying the behaviour of each butterfly visible at that point in time. Butterfly density was recorded (i.e., the number of butterflies visible in the frame) along with the sex of each individual (where possible). Individual behaviour was classified as either perching, patrolling, ovipositing, mate harassment and feeding, among others (see Table A1 and Figure A3 in Appendix 1). The scorer also scanned through the footage to count the number of pursuit flights that occurred during each filmed 15‐min period. We did not attempt to time the durations of these pursuit flights because individuals rarely stayed within the field of view for the entirety of their interaction.

We analysed the 15‐min video sequences of the two female dummies. The scorer recorded the time of the event in the video, which of the two models was approached and estimated the sex of the butterfly. If an individual was seen to approach both models in succession, only the first approach was recorded. In addition, the scorer timed the duration of interactions using the same criteria as used for the live butterfly interactions in the field.

### 2.6 Statistical Analysis

Data on male–female interactions were grouped into the hours of 7 am–9 am (hereafter, *early* morning) and 10 am–12 pm (*late* morning). We performed a Chi‐squared test of homogeneity to assess whether the frequencies of the observed male–female interactions (i.e., ‘approach/rejection’ and pursuit flight interactions) differed between early and late morning. For testing whether the duration of pursuit flights varied during morning hours, we performed a generalised linear model where flight duration was the dependent variable, time a continuous independent variable and field site a random variable with three levels. We analysed the durations that males interacted with the dummies during the morning hours using a generalised linear model with interaction duration as the dependent variable, time as a continuous independent variable and field site as a random variable with three levels. We acknowledge that repeated interactions may have occurred involving the same individuals, but due to the very high population densities at the study sites, we consider that the degree of pseudo‐replication would be low.

The duration of pursuit flight time and the times that males interacted with female dummies were normalised prior to analysis using the log‐transformation. Statistical analyses were performed using Statistica v7 (Statsoft, Tulsa, Oklahoma).

## 3 Results

### 3.1 Pupal Emergence Times

**FIGURE 1:**
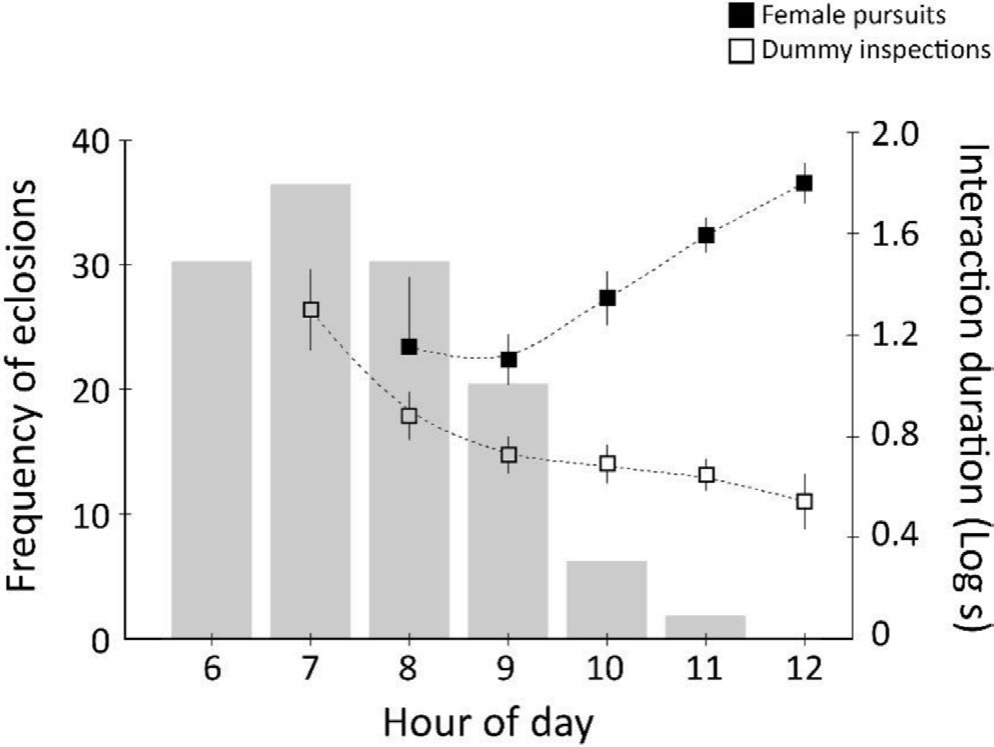
Frequency of eclosions per hour recorded in a glasshouse population of *E. hecabe* (columns) in relation to the courtship time investment of mate‐searching males (lines) recorded in the field. Solid squares represent the mean (±1 s.e.) duration that males spend in pursuit flight of evasive females (timed from direct observations at the field sites). Open squares represent the mean (±1 s.e.) duration that males interacted with perching female dummies across morning hours.

A total of, 141 adults emerged over the 2 days of pupal observations. As expected, butterflies began eclosing shortly after sunrise, with the peak of emergence occurring 1–2 h later (Figure 1). This finding confirmed our assumption with respect to the availability of freshly eclosed virgin females throughout the morning hours, thereby validating the rationale for our subsequent predictive tests regarding male behaviour.

### 3.2 Behavioural Observations and Dummy Experiments

**FIGURE 2:**
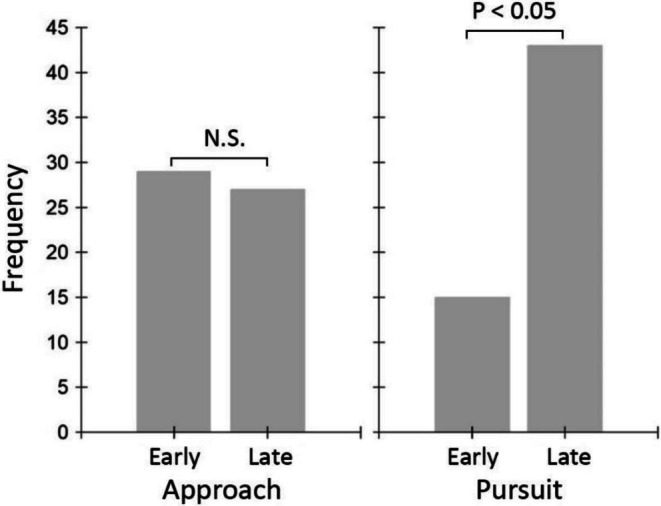
Frequency of male–female interactions scored from video observations of wild *E. hecabe* in the field in the early morning (‘Early’) versus late morning (‘Late’). ‘Approach’ refers to approach/rejection interactions; ‘Pursuit’ refers to pursuit flights.

**FIGURE 3:**
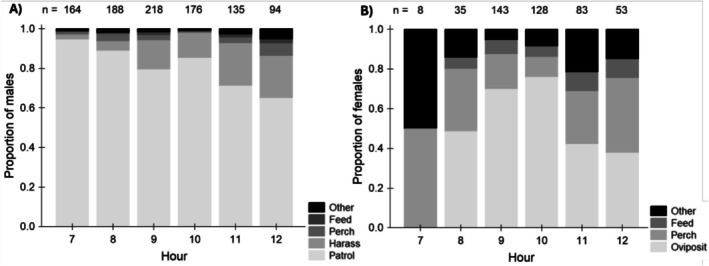
The breakdown of behaviours as observed for male (A) and female (B) *E. hecabe* across the morning hours. Data were collected from video recordings of butterfly behaviour at all three field sites over the five sampling days. Male harassment behaviour encompasses both approach/rejection and pursuit flight interactions. Sample sizes for each hour are given at the top of each column.

We scored 56 ‘approach/rejection’ interactions and 58 pursuit flights from the video footage recorded over 5 days. Whereas the frequency of ‘approach/rejection’ interactions remained the same across early and late mornings (Yates‐corrected *χ* ^2^_1_ = 0.0, *p* = 1.0), the frequency of pursuit flight interactions increased in late morning compared to early morning (Yates‐corrected *χ* ^2^_1_ = 6.19, *p* < 0.05; Figure 2). Mate harassment behaviour of males increased simultaneously with female ovipositing behaviour in the later morning hours (Figure 3A,B). Pursuit flight durations (*N* = 540, measured by an observer at a field site concurrently with video recordings of butterfly behaviour) increased across the morning hours (F_1,469_ = 41.6, *p* < 0.001) (Figure 1), and did not vary significantly among the different sites (F_2,469_ = 2.51, *p* = 0.082). Meanwhile, the time that males spent interacting with the dummies decreased across the morning hours (F_1,361_ = 22.15, *p* < 0.001), and did not vary significantly among the different sites (F_2,361_ = 2.34, *p* = 0.098).

## 4 Discussion

In this study we set out to test for theoretically optimal male mate search behaviour in a polyandrous, pupal mating butterfly. We hypothesised that male *E. hecabe* should shift between searching for teneral matings in the early mornings, and courting mature females in the late mornings, because this strategy would maximise their chances of copulation. We specifically predicted that the frequency of male–female interactions should shift from short inspective approaches in the early morning to pursuit flights in the late morning, and that pursuit flight durations should be longer in the late morning compared to the early morning. Overall, the evidence suggests that males do shift between the two tactics according to our hypothesis, but they do not completely cease the search for teneral matings even in the late mornings and continue to inspect perching butterflies even after the peak eclosion time has passed.

The behavioural shift is rather more subtle than seen in other butterfly species. For example, in *Coenonympha pamphilus*, *Chlosyne californica* and *Strymon melinus* , males switch from perching to patrolling behaviours on the basis of changes in factors such as ambient temperature (Wickman 1985) and male density (Alcock 1994; Alcock and O'Neill 1986). In a pupal mating butterfly *Heliconius sara*, males express flexibility in mate search strategies based on their size; large males jostle over access to the emerging female pupae, while small males, which are disadvantaged in jostling, establish and patrol territories to attain the rare unmated or receptive older females (Hernández and Benson 1998). Our results bear the greatest similarity to the work of Rutowski et al. (1988) on the polyandrous desert‐dwelling butterfly *Euphydryas chalcedona* . Rutowski et al. (1988) found that male mate search behaviour appeared to maximise encounters with non‐mated females that are easier to court compared to the already‐mated females. Males in this species also seemed to recognise mated females from non‐mated ones and expressed flexibility in how they invested in courting such individuals.

Our main hypothesis that males should employ two mate search tactics to maximise their likelihood of achieving matings was supported by most—but not all—of our observations. As would be expected if males were competing to locate teneral matings, they started patrolling early in the morning, which coincides with the peak eclosion times recorded in a glasshouse‐reared population (Figure 1). Males were also less likely to engage in pursuit flights in the early mornings, and the majority of interactions with non‐receptive females were of the short ‘approach/rejection’ type (Figure 2), which is consistent with a brief investigation to determine the presence/absence of a freshly eclosed female. By contrast, later in the mornings, males more frequently invested in significantly longer courtships with older, ovipositing females (Figures 1 and 2). This coincides with the timing of peak female ovipositing behaviour (Figure 3A,B). It is also likely the time at which most newly emerged butterflies have reached mobility. In fact, males kept consistently investing in longer pursuit flights towards noon, even though female ovipositing activity gradually decreased after peaking at 10 AM (Figure 1 and Figure 3B). We do not believe that temperature (i.e., a significant invertebrate mobility affecting factor in regions outside of the tropics) affected male likelihood to engage in pursuit flights, because temperatures were consistently relatively high (> 24°C from 7 AM) and males were readily flying actively.

Because time is a limiting factor in scramble competition for pupal matings, we hypothesised that males would spend little time harassing unreceptive females and invest in a rapid search of eclosing (or teneral) females in the early morning. Our dummy experiments do not support this, as males spent (slightly) more time courting the dummies in the early mornings (3.05 s) compared to late mornings (2.34 s) (Figure 1). We assume that males may have been encouraged to interact with the dummies as they didn't perform the classical reject behaviours (Shapiro 1970; Silberglied and Taylor 1978) of mobile females. It appears that males may invest more time in immobile targets in the early mornings when the likelihood of finding a virgin teneral female is greater.

We conclude that female reproductive biology determines the male mate search behaviour in the butterfly *E. hecabe* and that males shift between two different mate search strategies. Males invest in seeking teneral matings particularly during the early morning hours, when newly emerged females are available and pursue mature females later in the morning. Accordingly, in late mornings, males are more likely to engage in courting mobile females and spend longer durations in pursuit of females they encounter. Our observations on the ‘approach/rejection’ frequencies across morning hours and the frequency of inspections of female dummies in dummy experiments demonstrate that males do not stop the search for pupal mating even in late mornings. That may be because eclosion times have some variation, and late‐emerging individuals are relatively common (Silvasti, personal observation at the field sites and in the glasshouse‐reared population).

## Author Contributions

**Sanni A. Silvasti:** conceptualization (equal), data curation (equal), formal analysis (equal), investigation (equal), methodology (equal), visualization (equal), writing – original draft (equal). **Darrell J. Kemp:** conceptualization (equal), formal analysis (equal), funding acquisition (equal), methodology (equal), supervision (equal), validation (equal), visualization (equal), writing – review and editing (equal).

## Conflicts of Interest

The authors declare no conflicts of interest.

## Data Availability

The data presented and analysed in this manuscript are available in the Dryad Digital Repository: https://doi.org/10.5061/dryad.n5tb2rc6r.

## Appendix 1

**TABLE A1:** Description of butterfly behaviours observed in video scoring.

Behaviour	Description
Patrolling	Scrambling in the midst, or flying low at the edge of host plant vegetation
Ovipositing	Hovering flight near the tips of host plants where the female repeatedly lands on the leaves for 1–2 s to lay an egg
Perching	An individual spends time sitting in a resting position
Feeding	A butterfly approaches and lands on flowers for variable durations
Approach/rejection interaction	A butterfly approaches within 100 mm of another but ceases the courting attempt due to rejection behaviours
Pursuit flight	A male approaches a flying female or harasses a perching female into flight and then pursues her. Often escalates in a rapid ascending zig‐zag flight
Flyby	Prompt, often linear flight in and out of the camera field of view
Unknown	A behaviour that does not fit any of the above categories
